# Abscisic Acid: Hidden Architect of Root System Structure

**DOI:** 10.3390/plants4030548

**Published:** 2015-08-11

**Authors:** Jeanne M. Harris

**Affiliations:** Department of Plant Biology, University of Vermont, Burlington, VT 05401 USA; E-Mail: Jeanne.harris@uvm.edu; Tel.: +1-802-656-2933; Fax: +1-802-656-0440

**Keywords:** abscisic acid (ABA), root, meristem, development, lateral root, root architecture, developmental plasticity

## Abstract

Plants modulate root growth in response to changes in the local environment, guided by intrinsic developmental genetic programs. The hormone Abscisic Acid (ABA) mediates responses to different environmental factors, such as the presence of nitrate in the soil, water stress and salt, shaping the structure of the root system by regulating the production of lateral roots as well as controlling root elongation by modulating cell division and elongation. Curiously, ABA controls different aspects of root architecture in different plant species, perhaps providing some insight into the great diversity of root architecture in different plants, both from different taxa and from different environments. ABA is an ancient signaling pathway, acquired well before the diversification of land plants. Nonetheless, how this ancient signaling module is implemented or interacts within a larger signaling network appears to vary in different species. This review will examine the role of ABA in the control of root architecture, focusing on the regulation of lateral root formation in three plant species, *Arabidopsis thaliana*, *Medicago truncatula* and *Oryza sativa*. We will consider how the implementation of the ABA signaling module might be a target of natural selection, to help contribute to the diversity of root architecture in nature.

## 1. Introduction

Plants display an extraordinary diversity of form that distinguishes not only members of different taxa, but also individuals of the same species, even those that are genetically identical. This variation in form reveals differences in growth history.

Since most of plant development occurs after the plant escapes the confines of the seed, the environment has a major influence on the overall shape, or architecture of the plant. Thus, changes in the environment of a plant change its architecture, and this is why, although different individuals of the same species will look similar, none will have the same pattern of branching as another, either in the root or the shoot.

Patterns of growth are shaped by a changing and inconsistent environment. A walk through the woods demonstrates the strong effect that the distribution of light and shade has on the architecture of plants aboveground [1], but the environment belowground can be just as varied. Patches of nutrients or salts, physical obstacles, the presence of micro-organisms or other plant roots, and the availability of water all shape the architecture of the root system [2,3,4,5,6,7,8,9,10,11].

The architecture of the root system is determined by three factors: the position of branch roots (called lateral roots), the angle that they form with the parent root, and root length. The pattern of root branching and elongation, and thus the overall shape of the root system, is determined by a series of interactions of the root with its environment over the course of the plant’s lifetime.

Interactions with the environment require perception at the root surface and subsequent activation of a response within the root. Coordination of growth and development in different tissues and different regions of the root system in response to this environmental stimulus is usually mediated by hormones. All hormones have the ability to modulate root architecture at some level, and auxin, in particular, controls the function of root meristems, the patterning of tissues in the root tip, and the initiation of lateral roots [12,13]. Abscisic acid (ABA) is one of the most important hormonal mediators of abiotic and biotic signals, and as such, ABA acts as the interpreter of the environment, controlling key physiological processes such as germination, stomatal movements, dormancy and plant-microbe interactions [14]. Consequently, there is a growing awareness of the role of ABA in environmentally-regulated plant developmental processes, especially in the modulation of root architecture.

## 2. ABA Regulation of the Major Control Points of Root Growth: Cell Division and Cell Elongation

### 2.1. ABA Regulates Root Meristem Function

Although auxin is considered the primary hormone regulating root meristem function, ABA has been shown to modulate the major control points of root growth: cell division and cell elongation. Cell division in the root is restricted to meristems, regions that contain an organizing center (the Quiescent Center) surrounded by stem cells (initials) that divide to produce cells that differentiate into the various tissues of the root, as well as producing stem cells, that continue dividing and allow for indeterminate root growth [15,16]. Although high levels of ABA have long been known to inhibit root growth in well-watered conditions, analysis of the *Medicago truncatula latd* mutant indicated a positive role for ABA in the establishment or maintenance of root meristem function [17]. Subsequently, ABA was shown to regulate quiescence of the organizing center and inhibit differentiation in the *Arabidopsis* *thaliana* root meristem, thus maintaining the stem cell population [18]. Since the stem cells are the population of dividing cells, ABA regulates root growth by directly regulating the population of dividing cells in the root tip.

### 2.2. Modulation of Root Cell Length by ABA

ABA has been shown to also control root elongation by regulating cell length, which has a profound effect on root growth. Although continued cell division is essential for continued root growth, most of the very rapid root growth is driven by cell elongation of newly formed, differentiating root cells. In water-stressed maize plants, ABA stimulates the root elongation rate by increasing the length of the elongation zone and reducing reactive oxygen species (ROS) accumulation [19,20]. In *M. truncatula*, ABA plays a similar role, but in the absence of water stress, regulating cell length, and thus root elongation, by modulating levels of ROS via controlling expression of RESPIRATORY BURST OXIDASE HOMOLOG (RBOH) genes, encoding superoxide-generating NADPH oxidase enzymes [21]. Curiously, two nitrate transporters from the NITRATE TRANSPORTER 1/PEPTIDE TRANSPORTER (NPF) family [22], MtLATD/NIP (also known as MtNPF1.7) [22,23,24] and MtNPF6.8 regulate root cell length, and treatment with 10 μM ABA can override the effect of mutations in these genes to restore a more wild-type phenotype, either by increasing (for *latd* mutants) or decreasing (for nitrate-treated *npf6.8* RNAi mutants) root length [21,25]. For the *latd* mutant, it is clear that ABA restores root length in large part by reducing ROS accumulation to more wild-type levels and by increasing cell length [21]. This ability of the same concentration of ABA to modulate root length either up or down depending on the environmental conditions or genotypic background provides a nuanced way to fine-tune growth of the root system to provide the necessary plasticity to survive changing situations.

## 3. Control of Root Length by ABA

### 3.1. The Endodermis Is the Site of ABA Control of Root Elongation

Although ABA signaling occurs in all root cell layers, analysis from José Dinneny’s lab demonstrates that ABA signaling is required within the endodermis to regulate root growth [26,27]. By expressing the dominant *aba insensitive 1-1* (*abi1-1*) allele under different promoters to disrupt ABA signaling in specific root cell layers, they were able to determine that ABA signaling is required specifically within the endodermis for control of root elongation in response to salt both in primary roots and in lateral roots [26,27]. This finding is consistent with the observation by Ubeda-Thomas and colleagues that elongation of the endodermis is sufficient to control root elongation [28], and elevates the previously overlooked endodermis to a master role in the control of root growth. Within the context of salt stress, ABA can both stimulate growth of the primary root and inhibit growth of lateral roots by signaling within the endodermal layer [26,27]. This careful modulation of root growth by ABA in response to an environmental stimulus reveals a significant role for this hormone in shaping the overall structure of the root system.

### 3.2. Controlling the Transition from Proliferation to Differentiation

How does ABA control cell length? Also, is it coordinated with control of cell division? At this point, the detailed mechanism by which ABA controls these processes is still unclear. However, controlling the transition from proliferation to differentiation is a convenient way to coordinate cell division and cell length and is a likely target of ABA signaling. This transition is controlled by ROS molecules as well as by hormonal and genetic factors (discussed below). The regulation of ROS levels is a common signaling target for ABA in several plant species and in multiple tissues [19,21,29,30], and ROS molecules can function as a secondary messenger for both division and elongation, regulate gene expression and act directly on the cell wall, thus making it an attractive candidate for a mediator of cell length (reviewed in [31,32]). In *Arabidopsis*, ROS species control the transition between cell division and differentiation in the root apical meristem, thus modulating root length and providing an explicit link between cell division and cell length [33]. The common control of both meristem size (*i.e.*, number of cells in the division zone) and cell length by the KURZ UND KLEIN (KUK) F-box protein (AT1G60370.1), provides another mechanism by which both cell division and elongation can be coordinated [34]. Unfortunately, probes for the *KUK* gene were not present on the Affymetrix ATH1 array, so we know less about its regulation than that of other genes, but it is expressed in the cortex, endodermis and pericycle in the distal meristem and elongation zone, placing it in the same time and place as growth-regulating ABA signaling [26,34]. It will be interesting to see whether *KUK* expression or stability is a target of ABA signaling during regulation of root growth.

## 4. Lateral Root Development

### 4.1. Overview of Lateral Root Development

While root elongation is an important component of root architecture, the huge variety in the architecture of the roots of different plants is due largely to the formation of lateral roots both on the primary root as well as on lateral roots. By controlling the number and position of these lateral roots, the architecture of an individual plant can be sculpted to accommodate existing conditions and neighbors.

Lateral roots (LRs) form inside the primary root, initiating exclusively from divisions of the pericycle in plants such as *Arabidopsis* [35], and from a broader field of cells in other plants, but still centered around the pericycle, which plays the starring role. In legumes, LR development in white clover involves the cortex as well as the pericycle [36], and in soybean, *Lotus japonicus*, *Medicago truncatula*, and peanut, LR formation includes divisions in three tissue types: pericycle, cortex (sometimes multiple layers) and the endodermis [37,38,39,40]. A similar story is seen in *Curcurbita maxima* [37], and LR development in the monocots (rice, maize and *Allium cepa*) often involves cell division of both the pericycle and endodermis, but cortical divisions are also additionally observed in the formation of *Allium* lateral roots [41,42,43,44]. In Arabidopsis, prebranch sites are specified by oscillating cycles of gene expression in the meristem [45]. These prebranch sites have the potential to become LR founder cells, and some continue on to do so, dividing asymmetrically to form a LR primordium [35,45,46]. The developing LR emerges from the primary root by growing through the overlying layers of cells, which separate to allow the root to emerge [47]. Curiously, although the cells of the developing meristem are visible within unemerged *Arabidopsis* LR primordia, emergence is not the result of division of meristematic cells, but rather enlargement of cells at the primordium base [48]. It is only after emergence that the meristem becomes active, and cell divisions therein begin to drive growth of the root [48,49].

LR development can thus be broken down into several stages: initiation, or founder cell division, primordium development, emergence, and meristem activation. At this point, further elongation of the LR is controlled by the processes of cell division and cell elongation, as described earlier. While control of LR elongation has some distinct features that distinguish it from that of primary root development (for a discussion, see [26,50]), nonetheless, the basic parameters of growth are as described for the primary root.

The role of ABA signaling in LR development is intricately connected to environmental response, and, surprisingly, appears to regulate different aspects of LR development and have different effects in different plant species. Thus we will treat each topic in turn, so that we can treat ABA signaling in a developmental and phylogenetic context.

### 4.2. Different Developmental Roles for ABA in Different Taxa

The study of the role of ABA in LR development is confounded by the fact that its role varies in different plants. In a desire to understand something fundamental about LR formation, the field initially focused on understanding hormonal regulation in a single plant species, *Arabidopsis thaliana*. The huge variation in developmental patterns present in the natural world, however, suggests the existence of regulatory diversity. Thus, examining the role of ABA during this process of forming a new LR can allow us to begin to tease apart the rules (and exceptions!) that result in the diversity of root architecture present in nature.

Here, we will examine the role of ABA during LR development in three different plant species, *Arabidopsis thaliana*, *Medicago truncatula* and *Oryza sativa*.

#### 4.2.1. *Arabidopsis thaliana* 

**Figure 1:**
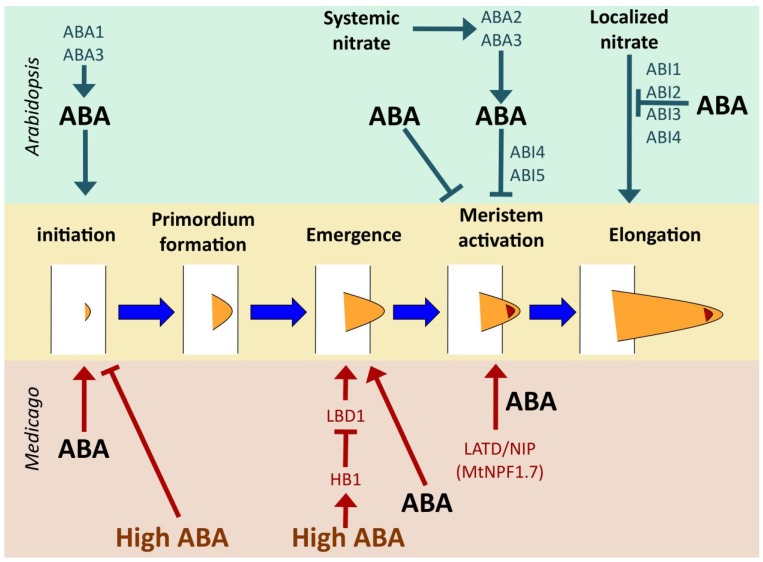
Regulation of lateral root development by ABA in *Arabidopsis* and *Medicago*. Key control points common to both species are initiation and meristem activation. Emergence from the primary root is an additional control point in Medicago. ABA stimulates initiation in both Arabidopsis and Medicago [55,56], but high concentrations of ABA inhibit it [56]. Salt stress inhibits lateral root emergence by stimulating ABA signaling, which induces expression of *HB1*, which in turn represses *LBD1*, required for emergence [56]. Lower levels of ABA stimulate lateral root emergence, but it is not known what functions downstream of ABA in this process [56]. Meristem activation is a key control point in both species, with systemic nitrate signaling via the classic ABA biosynthetic pathway in *Arabidopsis* and requiring *ABI4* and *ABI5*, but not other ABA signaling genes [54]. ABA treatment in the absence of an environmental nitrate signal also regulates meristem activation, but does not require activity of any of the known ABA signaling genes [57]. In *M. truncatula*, this step requires activity of the *MtLATD*/*NIP* (*MtNPF1.7*) gene [17,58]. ABA treatment can bypass the requirement for *MtLATD*/*NIP*, inducing meristem activation in the absence of gene function [17]. Elongation of the lateral root subsequent to meristem activation is regulated by localized nitrate in *Arabidopsis* and can be repressed by ABA signaling [54].

Auxin plays a primary role in the initiation of LR development, primordium formation and the subsequent passage through overlying cell layers, and there is a huge literature examining its function in these processes [12,51,52]. ABA, in contrast, appears to be largely limited to regulating the activation of the meristem once the LR has emerged from the parent root. In *Arabidopsis*, high levels of nitrate block the activation of the newly formed LR meristems, resulting in arrested, but emerged, LRs (Figure 1) [53]. ABA signaling mutants have increased stimulation of LR elongation by nitrate, indicating that ABA signaling plays an inhibitory role in LR elongation in Arabidopsis [54]. When the root system encounters localized nitrate (*i.e.*, a patch of nitrate), LR elongation is stimulated, in a process that is inhibited by ABA signaling (Figure 1). In both cases, ABA is involved in mediating an environmental signal (either high nitrate or localized nitrate) and affects later stages of LR development, meristem activation and LR elongation.

Several ABA signaling components have been implicated in LR development, but the way in which they interact to control LR formation is less clear. One of these components may be AtABI8/ELD1/KOBITO, a putative glycosyl transferase, with unknown substrates, that reduces seed and stomatal sensitivity to ABA (hence the ABA-insensitive designation), forms lateral roots that arrest immediately after emergence, and has defects in cellulose synthesis and cell elongation [59,60,61]. Although ABI8/ELD1/KOBITO has reduced sensitivity to ABA, its mechanistic relationship to ABA signaling is unknown. However, ABI8/ELD1/KOBITO has recently been shown to regulate plasmodesmatal permeability, suggesting that it may function downstream of ABA signaling to modulate root meristem function by controlling signaling between cells via plasmodesmata [62,63]. ABA transport likely also functions in the regulation of LR meristem activation, as loss of the ABA transporter, AtABCG40, results in a significant increase in LR formation in the presence of ABA [64]. The ABA receptor, PYR1-Like protein 8 (PYL8), drives LR meristem recovery after stress-induced arrest, but in a non-stress situation the LR phenotype of a pyl8 mutant is indistinguishable from that of wild-type [65]. Thus, the details of how ABA regulates the activation of LR meristems remain opaque, leaving room for further research in this area.

Mutants defective in ABA synthesis, which are impaired in the inhibition of LR elongation or meristem activation, not surprisingly have larger root systems both under standard growth conditions and when exposed to osmotic stress [54,66], revealing the overall inhibitory effect of ABA on LR formation in *Arabidopsis*. However, cutting the root tip off *Arabidopsis* ABA synthesis mutants (*aba1* and *aba3*) results in fewer LRs growing out from the parent root than wild-type [55]. This observation suggests that ABA must play a positive role in early steps of LR formation, probably initiation, since the authors rule out prebranch site selection [55]. Similarly, *aba2* mutants have reduced numbers of LRs when grown in soil, suggesting a stimulatory role for ABA at an early stage of development [67]. This positive role in LR initiation is subsequently masked by a more powerful inhibitory effect of ABA on LR elongation, leading to the overall larger size of the root system when ABA synthesis or signaling is defective, even though there is a mild stimulation of LR initiation [54,66]. Blocking carotenoid synthesis early in the pathway has a larger effect on the ability of the root to form LRs and cannot be completely rescued by adding back ABA, suggesting a role for an as yet uncharacterized carotenoid in addition to ABA [55].

#### 4.2.2. *Medicago truncatula* 

Although both *M. truncatula* and *Arabidopsis* share many aspects of life history and phylogenetics (both are annual, herbaceous eudicots from the Rosid clade), ABA appears to play a greater role in LR development in *M. truncatula* than in *Arabidopsis*. In *Arabidopsis*, ABA function appears to be largely limited to post-emergence LR development. In *M. truncatula*, however, ABA also regulates pre-emergence development, including LR initiation. Gonzalez and colleagues found that low levels of ABA (0.1 μM) increase the number of LR primordia, presumably due to increased initiation, although few early initiation events were observed, probably because they rapidly proceeded to the primordium stage [68]. Ariel and colleagues also observed a stimulation of LR initiation in response to 10 μM ABA (Figure 1) [56]. In addition, they found that the percentage of emerged lateral roots when considered out of the total number of initiated lateral roots was significantly increased in response to 10 μM ABA, indicating that ABA also stimulates the process of emergence, not just initiation (Figure 1). However, higher levels of ABA have an inhibitory effect, similar to the effect of high salt, on both LR initiation and emergence, indicating a more nuanced response, with the concentration of ABA determining the outcome, similar to the biphasic response model proposed for ethylene signaling [56,69]. Analysis of the *M. truncatula* *latd* (Mt*npf1.7*) mutant revealed a role for ABA also in LR meristem activation. *latd* lateral roots arrest immediately after emergence from the primary root; ABA treatment activates the meristem, permitting elongation of the lateral root [17]. Not surprisingly, the stimulation of three earlier developmental steps (initiation, emergence and meristem activation) translates into a significant increase in the density of elongating LRs in response to ABA treatment at a variety of concentrations [17,56,68,70].

Perhaps, the most interesting difference between ABA regulation of LR development in *Arabidopsis* and *M. truncatula* is that while ABA signaling largely inhibits LR development in *Arabidopsis* (1 μM ABA is sufficient to block the development of visible LRs), ABA primarily plays a positive role in *M. truncatula* LR development, stimulating initiation, primordium formation, emergence and meristem activation at concentrations ranging from 0.1–10 μM and only inhibiting LR formation at 50 μM [56,68,70]. This stimulation of LR production by ABA is observed in multiple legume species, such as *M. sativa*, *Lotus japonicus*, *Lupinus sericeus*, *Mimosa pudica*, *Senna hebecarpa*, and *Cladastris lutea*, as well as in the non-legume, actinorhizal nodulator, *Casuarina glauca* [56,70]. These observations are consistent with phenotypes of the *Lotus japonicas* ABA-insensitive mutant, *beyma*, and the ABA-deficient mutant, *enhanced nitrogen fixation 1 (enf1)*, both of which have reduced numbers of LRs under standard growth conditions, supporting the idea that ABA stimulates LR development in legumes [71,72]. Exceptions include the basal legumes, *Cercis candensis* and *Chamaecrista fasciculata,* which show neither an increase nor a decrease in LR density, as well as garden pea (*Pisum sativum*) and peanut, in which LR formation is inhibited, although LR density was not assessed in peanut, making it difficult to know whether the decrease in LR number was due simply to decreased primary root length [70,73,74]. Other than *Casuarina glauca*, all non-legumes tested showed a decrease in LR density in response to ABA, but one: rice [70,75].

#### 4.2.3. *Oryza sativa* 

The structure of the grass root system has significant differences from that of eudicots like *Arabidopsis* and *M. truncatula*. In addition to the seminal root that comes from the seed, *O. sativa* (rice) forms adventitious roots, called nodal, or crown roots, which form on the stem, postembryonically [51,76]. All of these roots can themselves form lateral roots [76]. Much is known about the role of ABA in rice LR formation, but there are some interesting hints. As it does in *M. truncatula* and most legumes, ABA treatment promotes LR production in rice, stimulating lateral root initiation in seminal roots in a process that requires calcium, calmodulin and protein synthesis [75]. ABA also inhibits emergence of crown roots from the nodes of the stem, counteracting the effect of GA [77]. Although these roots are adventitious roots, rather than lateral roots, they will function as a major part of the rice root system, forming lateral roots themselves. It will be interesting to learn whether ABA also promotes LR emergence and meristem activation in rice, as it does in *M. truncatula*, or rather plays an inhibitory role, blocking meristem activation as it does in *Arabidopsis*. Also, it is possible that the different types of roots have different responses to ABA, just as they have different growth patterns and functions in the root system [78].

## 5. ABA Shapes Root Architecture in Response to Environmental Signals

The soil environment is very heterogeneous, with nutrients and salts present in an uneven distribution that is caused by changes in the environment as well as the action of microbes. Plants have evolved strategies to respond to these local changes in the root environment by restricting or promoting growth, and, in particular, by regulating the formation or outgrowth of LRs.

### 5.1. Regulation of Root Growth and Branching in Response to Abiotic Stress

One of the classic functions of ABA is to mediate plant responses to abiotic stress, and one of the ways roots respond to stress is by altering root architecture, changing patterns of growth and quiescence. A major abiotic stress for the root system is lack of water, which is likely to be patchy, with some areas of soil having more water and some less [79]. Plants respond to heterogeneous soil moisture, locally altering ABA levels in response to different amounts of water [79]. Transpiration draws preferentially from well-watered roots, redistributing water throughout the plant [80,81]. Thus, ABA levels respond not only to local water availability, but also to overall water levels experienced by the root system as a whole [79]. Consequently, changes in the root environment will have both local and systemic effects on ABA-mediated responses.

Drought stress is related to salt stress and osmotic stress and ABA is an important mediator of all three. While all three reduce soil water potential, salt stress has the added component of ionic stress, and both salt stress and osmotic stress have increased osmotic strength.

The effect of drought stress on root architecture is most directly assessed by growing plants on media of different water potential, for example by growing plants on vermiculite to which different amounts of water have been added. This approach has been used very effectively to find that ABA synthesis is required for maize plants to maintain root elongation under conditions of water stress (water potentials of −1.6 MPa) [20,82]. Similarly, both millet and wheat nodal roots have stimulated root elongation in water stress, but the role of ABA in mediating this response is unknown [3,83].

The effect of salt stress on root architecture has been the focus of more studies than that of any other ABA-mediated abiotic stress, because it is easier to manipulate and control. Salt signals through ABA, but not exclusively, signaling also through other, ABA-independent pathways [84]. In this review, we will examine only salt-regulated changes in root architecture that are known to operate via ABA signaling. Our understanding of the connection between salt, ABA and root architecture has been enhanced by a series of elegant experiments on *Arabidopsis* from José Dinneny’s lab, demonstrating that ABA has differing effects on lateral and primary roots, with LRs exhibiting much greater sensitivity to exogenous ABA and to salt treatment [26]. The end result is that salt, signaling via ABA, shapes the root system by inducing a period of quiescence in newly emerged LRs, perhaps equivalent to the block to LR meristem activation described in nitrate-regulation of LR development, followed eventually by resumption of growth [26,54]. In Arabidopsis primary roots, ABA promotes growth recovery after a short period of salt-induced quiescence [27], similar to what has been seen in maize, which responds to salt stress with an increase in primary root elongation. Recently, Zhao and colleagues have found that ABA signaling is also required for elongation during the recovery phase of LR growth, following stress-induced quiescence, indicating a role for ABA both in growth quiescence and in recovery of the same root organ [65]. In *M. truncatula*, high salt reduces LR initiation and stimulates expression of the MtHB1 transcription factor to repress LR emergence [56].

One difficulty in comparing various studies on salt regulation of root architecture is that the starting concentrations of salts vary greatly, from the high salt concentrations in Murashige and Skoog (MS) medium (commonly used for *Arabidopsis*) to the much lower salt concentrations in Fahräeus medium (often used for *Medicago*) [85,86]. There are, of course, many salts, but salt stress studies usually focus on NaCl-induced stress. Concentrations of other salts, however, are also relevant. For example, increased potassium levels protect against NaCl stress [87], and potassium concentrations can vary from 20 mM in full-strength MS medium to 0.7 mM in Fahräeus, thus potentially modulating the effect of the NaCl treatment on plant development [85,86]. Differences in salt concentration will also affect the osmotic strength of the medium, and this may explain why *Arabidopsis* LR initiation is greater on 0.2× MS medium than 1×, although this difference could also be due to changes in nitrogen or the nitrogen: carbon ratio of the media [88]. Thus, having a consistent starting point, or controlling for osmotic effects to determine whether a salt effect is due to the salt itself, or rather to the changed osmotic potential, would make it easier to compare studies done in different labs and with different taxa.

The role of osmotic stress in root architecture is less clear, and has been hard to test. Generally, regulation of root growth by water availability is tested by changing the osmotic concentration of the media. Certainly, plant roots exhibit hydrotropism (growth towards water), which structures the root system [89]; however, the way in which the process is tested can affect the outcome. MacGregor and colleagues have found that when *Arabidopsis* plants are grown on agar plates containing sucrose, contact of the shoot with the sucrose-containing medium stimulates LR formation under mild osmotic stress [90]. ABA is required for the osmotic signal from the roots to increase permeability of the shoot to sucrose, resulting in an increase in LR emergence [90]. Thus, assays involving growth on sucrose-containing medium in which shoots contact the medium are difficult to interpret, and may explain the opposite LR phenotypes of Arabidopsis *aba2* mutants when grown on sucrose-containing agar plates (more LRs) and when grown in soil (fewer LRs), although this could be due indirectly to small shoot size, resulting in reduced resource allocation to the roots. [66,67]. However, ABA also regulates LR formation even when Arabidopsis plants are grown on medium lacking any carbon source [26,70], so stimulation of a shoot sucrose-sensing pathway cannot be the only mechanism by which ABA regulates LR development. It will be interesting to test the role of osmotic stress in the regulation of LR development in a system in which interactions are limited to the root and its growth medium.

### 5.2. Hydrotropism, Halotropism and Water Patterning

Even under non-stress conditions, the presence or lack of water and salt patterns the growth of the root system. Hydrotropism, growth towards water, guides roots across the heterogeneous soil environment towards areas of more water (higher water potential) [89]. Halotropism is the growth of roots away from areas of high salt concentration [91]. Since this growth process is also driven by water potential gradients, sometimes created using salts, hydrotropism and halotropism area really two sides of the same coin. Both of these processes involve root elongation in a particular direction, either towards high water potential (hydrotropism) or away from low water potential (halotropism). However, if the stimulus is really just water potential, then these may be the same physiological process, studied with different tools. Hydropatterning is the formation of LRs in a particular position on the primary root, influenced by patterns of water availability (*i.e.*, water potential).

The process of hydrotropism requires ABA signaling; mutants defective in ABA synthesis or ABA signaling have a reduced hydrotropic response. Mutants defective in gravitropism have an increased hydrotropic response [92] and the *no hydrotropic response 1 (nhr1)* mutant, which was identified based on its reduced hydrotropic response, has an increased gravitropic response [93]. Thus, increased hydrotropism involves the simultaneous inhibition of gravitropism, allowing the pursuit of water to dominate root growth at the expense of growth in the direction of the gravity vector. Recent analysis by Antoni and colleagues demonstrate the involvement of the core ABA signaling module including the PYR/PYL/RCAR ABA receptors and the PP2C co-receptors [94]. They found that lines lacking six PYR/PYL/RCAR ABA receptors had a strongly reduced hydrotropic response, whereas a line lacking 4 PP2C co-receptors had an enhanced hydrotropic response [94]. Curiously, although both salt signaling and hydrotropism require or are affected by ABA, the link between halotropism and ABA has yet to be explored. Hydropatterning, however, appears not to require ABA, and ABA signaling mutants, including *abi2-1* and the *pyr*/*pyl 112458* sextuple mutant, exhibit normal hydropatterning [2].

### 5.3. Responses to Nitrate: NPF Transporters Connect Nitrate, Hormones and Root Architecture

Plants respond to patches of nitrate by locally stimulating growth of lateral roots to exploit the concentration of nutrients, a foraging behavior mediated both by auxin and by ABA. In Arabidopsis, local changes in rhizosphere nitrate concentration are sensed by the bifunctional AtNRT1.1 (AtNPF6.3) nitrate sensor/auxin transporter in the root tip [95,96]. Nitrate competes for auxin transport by AtNRT1.1, thus changing the accumulation of root tip auxin in a nitrate patch and altering root growth to locally modulate root system structure [95]. Although the mechanism is unclear, we know that ABA negatively regulates this foraging behavior, because both ABA synthesis and signaling mutants have greatly stimulated LR elongation in nitrate patches (Figure 1) [54]. In contrast, when roots experience high nitrate levels throughout their root system, rather than just in a patch, LR elongation is repressed. This repression, also mediated by ABA, inhibits meristem activation, thus pausing the LRs until growth is desired (Figure 1) [54]. Recently, Léran and colleagues have shown that the protein phosphatase ABI2 physically interacts with AtNRT1.1/AtNPF6.3, relieving repression by a complex formed by the CIPK23 kinase and the CBL9 calcium sensor to allow nitrate transport [97]. ABI2 is an ABA co-receptor, interacting with the PYR/PYL/RCAR proteins upon ABA binding, which inactivates the ABI2 phosphatase activity [98,99]. Thus, ABA represses nitrate uptake by AtNRT1.1/AtNPF6.3 by inactivating ABI2, providing a mechanism to reduce energy-consuming nitrate assimilation processes during stress conditions [97]. AtNRT1.1/AtNPF6.3 also transports auxin, and this activity is competed by transport of nitrate [95]. It will be interesting to learn whether ABI2 also regulates the auxin transport activity of this nitrate transporter to thus modulate root architecture.

The *M. truncatula* nitrate transporter LATD/NIP (MtNPF1.7) [22,23,24] is required for activation of LR meristems, and the lack of a functional *LATD*/*NIP* gene can be compensated for by addition of ABA to the root system, allowing newly emerged LRs to form a functional meristem and grow (Figure 1) [17,58]. In *M. truncatula*, systemic nitrate inhibits primary root elongation and reduces LR density in wild-type plants, but *latd* roots are already short and do not exhibit any further decrease in root length or LR density in response to nitrate treatment [23]. Interestingly, *MtLATD*/*NIP* expression is not significantly altered by a 2-hour nitrate treatment, but it is regulated by the hormones ABA, auxin and cytokinin, and is expressed most strongly in the root tip, suggesting an important role in hormonal responses in the growing root [23]. *MtLATD*/*NIP* is also strongly expressed in nodule meristems and is required for the function of that meristem after emergence of the symbiotic organ from the host root, in a developmental process that shares many similarities with that of lateral roots, suggesting a common evolutionary origin [23,58].

The NPF transporter, MtNPF6.8, is a dual affinity nitrate transporter that regulates root growth by also transporting ABA, a mechanism that strikingly parallels the function of AtNRT1.1 (AtNPF6.3), an NPF family member from the same subclade [22,25]. Mutants of *MtNPF6.8* are insensitive to the inhibition of root growth by nitrate, and have longer cells, indistinguishable from those of plants grown on nitrogen-free medium. ABA treatment rescues this mutant phenotype, restoring wild-type cell length in the presence of nitrate [25]. An *MtNPF6.8* promoter-GUS fusion is expressed in the root tip and in LR primordia and expression is stimulated by ABA, but not nitrate [25]. *MtNPF6.8* acts by regulating root elongation (Figure 1), but its expression in LR primordia suggests a possible role in some stage of LR development as well. It is tempting to speculate that MtNPF6.8 might function as a nitrate sensor, just as AtNPF6.3 does in Arabidopsis roots, and coordinate nitrate sensing with root growth, both through control of elongation and of LR formation, via ABA transport.

Yet another NPF family member, AtNPF4.6/AIT1/NRT1.2, this one from subclade 4, provides another connection between ABA and nitrate in the growing root system, but the biological link between these two processes is unclear. AtNPF4.6 is expressed in roots and transports both ABA and nitrate [100,101], but nitrate has no effect on ABA transport, unlike the situation for AtNRT1.1 (AtNPF6.3) [102]. Also, the affinity of AtNPF4.6 for ABA is several orders of magnitude higher than for nitrate, indicating that ABA transport may be its primary function [100]. The effect of ABA on nitrate transport was not tested, but it is less clear what the biological purpose of this would be. Unlike the other three NPF transporters discussed here, AtNPF4.6 is expressed in the mature root, but not in the growing root tip, suggesting that it may not be involved in elaboration of root architecture, but rather in long-distance transport of nitrate and ABA throughout the plant [100].

## 6. ABA Functions in a Hormone Signaling Network

Like all plant hormones, ABA functions as a part of a much larger hormone signaling network [103]. As a result, any changes in ABA levels or signaling strength will echo through other hormonal pathways. In particular, ABA exerts at least some of its effects on root growth by influencing ethylene, gibberellin (GA) and auxin signaling. In maize a key function of ABA during drought conditions is to inhibit ethylene synthesis, which inhibits root growth, thus promoting root elongation (by inhibiting an inhibitor) to potentially tap into deeper water supplies [82]. A similar story has been observed in *Arabidopsis* roots, in which ethylene signaling functions downstream of ABA under normal growth conditions to inhibit elongation [104,105], and this linear pathway intersects with auxin signaling [106], discussed below. In salt signaling, GA and ABA have opposing effects on root growth, in part by their opposing effects on the growth-inhibiting DELLA proteins [107,108]. However, this antagonistic interaction only gives half the story, because ABA also regulates root growth independently of DELLA proteins and the salt-stimulated increase in DELLA levels will occur even in the absence of ABA signaling [26].

**Figure 2:**
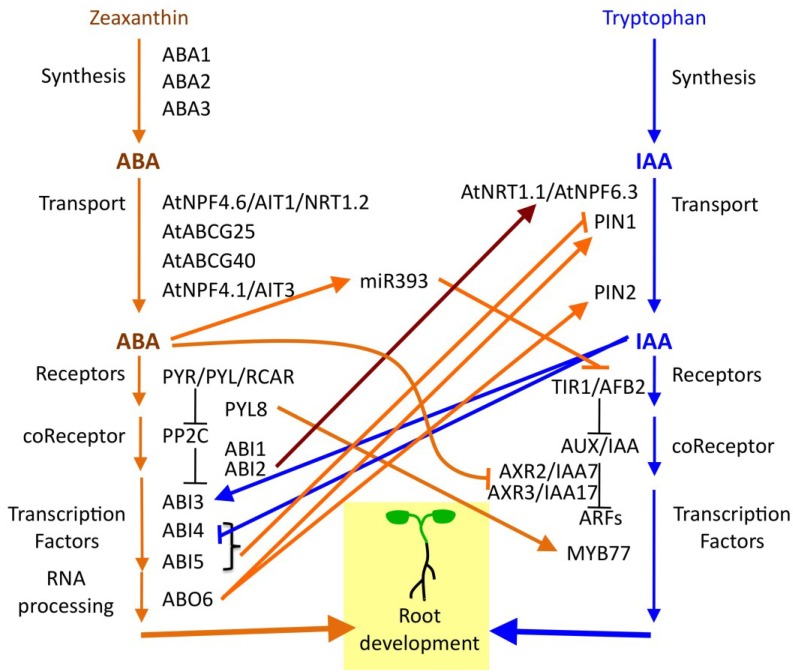
Interactions between ABA and auxin signaling pathways during elaboration of the Arabidopsis root system. This diagram is restricted to genes mentioned in the text and does not include all ABA or auxin signaling genes. Downward arrows do not imply a simple linear pathway, but are rather used to group genes according to function. Crosstalk between the ABA and auxin signaling pathways are indicated by arrows, signifying a positive interaction, and blocked lines, signifying a negative interaction. When evidence for crosstalk is based on treatment with exogenous ABA or IAA, and a specific gene mediating this activity is unknown, the arrows originate from the hormone name, rather than a gene name. Orange arrows indicate interactions originating from the ABA pathway. Blue arrows indicate connections originating from the Auxin pathway. The red arrow indicates a connection between ABA and Auxin signaling components that may be independent of their function in ABA or Auxin signaling. ABI2 regulates the nitrate transport function of AtNRT1.1/AtNPF6.3, but its effect on auxin transport is unknown [97]. AtRAC7/ROP9 is not included in this diagram, although it is clearly involved in ABA/Auxin crosstalk, because its role connecting these pathways may be context dependent [109,110].

Since auxin is a major regulator of development in general and is involved in patterning the root tip and controlling initiation of LR development, there are many complex interactions between auxin and ABA signaling in the elaboration of the root system. Auxin moves through the plant by polar auxin transport. Direction of auxin flow is determined largely by the position of auxin efflux transporters on the cell’s plasma membrane. One important point of crosstalk between ABA and auxin signaling, is regulation of polar auxin transport by ABA, which targets both the PIN-FORMED 1 (PIN1) and PIN2 auxin efflux transporters to inhibit root elongation in *Arabidopsis* (Figure 2) [30,106,111,112]. This process requires the transcription factors ABA-INSENSITIVE 4 (ABI4) and ABI5, which function in the core ABA signaling pathway [111,112], as well as the ABA-OVERLY SENSITIVE 6 (ABO6) protein required for mitochondrial splicing [30], all of which also regulate PIN1 expression in the root tip (Figure 2). The change in PIN1 expression alters root auxin transport [112] and modulates the size of the root meristem [111]. Lowering PIN1 expression will also lower auxin accumulation in those cells, and thus expression of several auxin-responsive genes is also reduced [30,112].

ABA can both repress and stimulate root growth [17,19,26,27,67], depending on environmental and developmental context, root type, genotype and species, thus it is not surprising that ABA signaling can both stimulate expression of some genes typically considered to be auxin-responsive as well repress others. In *Arabidopsis*, ABA treatment represses growth of the early seedling by altering auxin signaling by reducing transcript levels of AXR2/IAA7 and AXR3/IAA17 (Figure 2) [113]. ABA also induces expression of the microRNA, miR393, which cleaves transcripts for the auxin receptors, TIR1 and AFB2, thus reducing auxin signaling and inhibiting LR growth (Figure 2) [114]. As described above, wild-type function of ABO6 is required to induce expression of PIN1 and PIN2 in the root tip, and thus promote auxin transport to stimulate auxin-responsive gene expression [30]. Sometimes this crosstalk between ABA and auxin signaling is more context-dependent, as when a single ABA receptor, PYL8, physically interacts with transcription factor MYB77 to stimulate expression of auxin-responsive genes, thus promoting LR growth specifically during the recovery phase following stress-induced quiescence (Figure 2) [65]. Other crosstalk is more labile, suggesting the existence of additional, as yet unknown, environmental factors that may modulate the interaction between these two hormones in a complex network. For example, the AtRAC7/ROP9 protein is a modulator of both auxin and ABA signaling, but depending on growth and assay conditions, or perhaps level of expression, different studies have found that it can either stimulate or repress ABA and auxin responses [109,110]. Of course, auxin signaling modulates this entire process by controlling expression of key regulatory nodes, inducing expression of ABI3 in LR primordia and inhibiting expression of ABI4 [112,115].

There is clearly extensive interplay between auxin and ABA signaling during root growth in plants other than Arabidopsis, but at this point we just have a few vignettes. The synthetic DR5 promoter [116] is now a classic tool for reporting auxin responses, although in the Arabidopsis root tip it also responds to regular oscillations of gene expression modulated by auxin signaling [45,46]. DR5 reporter fusions are used to identify sites of LR initiation and small primordia, and, as such, reflect response to auxin signaling. Addition of ABA affects expression of DR5 reporters as expected: in peanut, where ABA inhibits LR initiation, ABA represses expression of DR5:GUS in LR primordia as well as that of an AUX1 homolog [74]; in *M. truncatula*, where ABA stimulates LR initiation, ABA treatment increases the number of sites expressing a DR5: Venus reporter, reflecting the increased number of LR primordia [68]. In rice, the ABI5-LIKE1 (ABL1) gene negatively regulates auxin signaling, but is itself auxin-inducible, revealing a negative feedback loop in which auxin represses its own signaling [117]. Thus, root growth in *abl1* mutants is hypersensitive to inhibition by auxin [117]. One of the most interesting findings is the identification of the *M. truncatula Homeobox 1* (*MtHB1*) gene that modulates root architecture by controlling an auxin-mediated developmental pathway [56]. MtHB1 is a member of the HD-ZIP family of transcription factors and is strongly induced by short-term salt or osmotic stress and by ABA in root tips, where it binds the promoter of the *LOB-Binding Domain 1 (LBD1)* gene and represses its expression, blocking lateral root emergence [56]. *MtLBD1* is associated with early stages of LR development and is auxin-inducible. Thus, in this case, ABA is signaling through *MtHB1* to repress auxin-inducible *MtLBD1* regulation of early LR development [56]. Interestingly, LBD transcription factors are associated both with LR initiation in Arabidopsis and with the root tip oscillations of gene expression that lead to prebranch point initiation (many of which subsequently form LRs) [45,118], suggesting that ABA may intersect with this conserved signaling module to regulate developmental plasticity in response to environmental stress.

## 7. Diversity of Root Architecture in Different Species: ABA Signaling as a Target of Evolution?

Plants elaborate a great variety of root structures as a result of their interaction with the environment, but also their genetic make-up. ABA signaling appears to be an ancient acquisition, with the pathway well established prior to the diversification of land plants [119,120]. Nonetheless, different plant species appear to have different root architecture responses to ABA [57,70,75] and ABA controls different aspects of LR development in different taxa, primarily regulating post-emergence development in Arabidopsis [54,57] and earlier stages, such as initiation in *M. truncatula* and rice [56,75] and primordium development and emergence in *M. truncatula* [56,68].

How might ABA control different aspects of LR development in different plant species? The core ABA signaling pathway is highly conserved within the angiosperms, so it seems most likely that this signaling module has remained intact, and is being deployed at different times or with different targets. Alternatively, it may be that the neighborhood of relationships within the larger signaling network in which ABA signaling functions has been subtly altered. ABA often functions in a signaling network with ethylene, and that has been proposed to be a major way in which ABA regulates root elongation. However, in different plant species ABA signaling is positioned differently relative to ethylene, functioning upstream of ethylene in maize and Arabidopsis in the regulation of root growth [82,106,121] and downstream of ethylene in *M. truncatula* and rice roots [17,122]. Both *M. truncatula* and rice respond positively to exogenous ABA by stimulating LR initiation; perhaps different relationships between ABA and ethylene make possible different regulation of root growth in the elaboration of the root system of different plants.

The movement of ABA is vital to its function in coordinating environmental responses between distant, or even neighboring, plant tissues. The coincidence of a major QTL for root growth and the gene for the ABA transporter, Mt*NPF6.8*, in *M. truncatula*, suggests that variation in ABA transport can be a source of natural diversity for root length [25]. Mt*NPF6.8* is also expressed throughout LR primordia and later in the tip of growing LRs [25]; perhaps variation in ABA transport can also modulate LR development and thus alter root architecture? Curiously, transport or exudation of ABA from rice roots during severe water stress is essential for adaptation of the plant to this stress, and reduced accumulation of ABA in the root of upland rice is associated with increased root biomass and drought tolerance [123]. Most plants form an exodermis around internal root layers that prevents the leakage of ABA, among other things, into the rhizosphere. Under conditions of severe water stress, roots of most plants accumulate high levels of ABA. Upland rice, however, is able to release that ABA into the rhizosphere because under severe water stress their roots do not form a Casparian band in the schlerenchyma, thus permitting ABA exudation into the rhizosphere and lowering root ABA levels [123]. The fact that the plant reserves ABA exudation for severe water stress conditions and that not all rice varieties have this ability indicates that the process is regulated and is also under selection. Curiously, legumes are among the few plant taxa that lack an exodermis and thus leak ABA into the rhizosphere [124]. Perhaps, this loss of ABA from legume roots is not completely uncontrolled, but actually functions more like an overflow valve, as it does in drought-tolerant upland rice. Alternatively, this could provide a mechanism to create a gradient of ABA in legume roots or perhaps generate higher levels in outer root cell layers, as ABA moves from the stele towards the rhizosphere, providing another mechanism for regulating root architecture.

## 8. Conclusions

Clearly, the diversity of strategies used by plants to alter ABA levels or move ABA within or out of the plant provides a likely target for natural selection to act upon to generate the huge variety of plant forms. With increased attention being paid to genetic and environmental control of root architecture in a variety of species, we will begin to unravel the various ways that the ancient ABA signaling module has been implemented to modulate root growth and control developmental plasticity. Such a comparative physiological approach will help us to better understand the evolutionary and environmental constraints that shape root architecture and have a better appreciation for the varied developmental pathways responsible for building the root system.

## 9. Future Avenues

Despite strong progress, several open questions remain.

Multiple Seasons: One big limitation of the current studies is that the three major plant species examined, Arabidopsis, *M. truncatula* and cultivated rice, are all annual, herbaceous plants. Perennial plants must contend with changing seasons and variations in environment from year to year. Since land plants are rooted to the spot where they germinate, they must respond to changes in the environment by altering growth and physiology. The architecture of the root system is a fixed record of the history of the growth of that plant. How do varying seasons and environmental conditions affect root growth in a perennial plant? What role does ABA, an important mediator of environmental conditions, play in this process? And a related question: How are things changed or the same in woody plants?

Biotic interactions: Biotic interactions play a significant role in shaping the plant root system. Many microbes make or catabolize ABA [125,126,127,128,129]. What is the role of microbially-synthesized ABA in shaping the root system? Does ABA “leaking” out of the root signal root-associated microbes? Does selective degradation of ABA by microbes in different parts of the rhizosphere guide root growth?

Plant-Plant competition: Some plants exude ABA, others do not. LR formation is stimulated by ABA in some plants and not in others. What happens when roots of a non-legume grow past the roots of a legume or upland rice plant that exudes ABA? Is this one strategy plants use to inhibit root growth of competitors in their root space?

Other Environmental Signals: Finally, what other aspects of a plant’s environment are mediated by ABA signaling and shape root architecture? Is there a role for ABA signaling in the altered branching response to nutrients such as phosphate or iron?

It will be interesting to see what the function of ABA is in controlling root growth in these different situations. Comparative physiology of ABA in root development may be the key to unlock many of the secrets of plant diversity both in environmental response and in genetic control of root architecture.
